# A Mass Spectrometric Study on Tannin Degradation within Dyed Woolen Yarns

**DOI:** 10.3390/molecules24122318

**Published:** 2019-06-22

**Authors:** Ilaria Degano, Marco Mattonai, Francesca Sabatini, Maria Perla Colombini

**Affiliations:** Dipartimento di Chimica e Chimica Industriale, Università di Pisa, via Moruzzi 13, 56124 Pisa, Italy; mmattonai@gmail.com (M.M.); f.sabatini4@gmail.com (F.S.); maria.perla.colombini@unipi.it (M.P.C.)

**Keywords:** natural tannins, polyphenols, high-resolution mass spectrometry, oak gallnut, *Juglans regia*, *Acacia catechu*

## Abstract

Natural tannins from various plants have been used throughout human history in textile dyeing, often as mordant dyes. The ageing behavior of these dyes is a challenge in conservation science, requiring a thorough knowledge of the textile–mordant-dye system. In this work, we analyzed reference wool yarns dyed with natural tannins from oak gallnuts, walnut (*Juglans regia*), and catechu (*Acacia catechu*), after artificial ageing. To gain insights on the composition of the dyestuffs and on how they aged, an analytical procedure based on extraction with Na_2_EDTA/DMF (ethylenediaminetetraacetic acid/dimethylformamide) and high-performance liquid chromatography (HPLC) analysis using high-resolution mass spectrometry detection was used. Since conventional reversed-phase (RP) columns usually show poor retention efficiency of highly polar compounds such as tannins, an RP-amide embedded polar group stationary phase was used to achieve optimal retention of the most polar compounds. Tannins from oak gallnuts showed little degradation after ageing, while a significant increase in the content of hydroxybenzoic acids was observed for tannins from walnut and catechu. Finally, the analytical procedure was applied to characterize the tannin dyes in historical tapestries from the 15^th^ to 16^th^ century, and the results were discussed in comparison with the reference yarns.

## 1. Introduction

Tannins are polymeric polyphenols. They are a group of secondary plant metabolites whose primary biological function is protection against bacterial and fungal attacks. Thousands of different polyphenols have been identified throughout the vegetal kingdom [1], resulting in a high number of possible structures of tannins, which makes it challenging to define a universal classification method. The most widely accepted classification divides tannins into two main categories [2,3]. The first is constituted by hydrolysable tannins, which are the most common in woody plants. They are composed of gallic acid and ellagic acid units linked to monosaccharides by ester bonds. The second category consists of condensed tannins, which are made of flavonoid units linked by C–C bonds.

Tannins have been used for various purposes throughout history, thanks to their nutraceutical properties and their ability to convert animal skin into leather [4,5,6,7]. One of the most traditional uses of tannins is as natural dyes or adjuvants in dyeing, providing a wide range of shades going from pale yellow to dark brown [8]. Wool and silk clothes, tapestries, and carpets colored with tannin-based dyes can be found in many cultures and eras of human history [9,10,11,12]. They can also be used in the weighting process for silk, after degumming [13]. Tannins are classified as mordant dyes, as their durability on textiles is significantly improved using metallic ion mordants such as aluminum and iron [14].

The most widely used source of tannins is gallnuts, which are formed from various plant species as a response to insect infestation, and are composed of about 75% of hydrolysable tannins [15]. The first reference to a tannin and mordant-based coloring material is relative to iron-gall inks, cited in the *Naturalis Historia* by Pliny the Elder [16]. Other common sources of tannins are the bark and fruits of plants from the *Juglans* genus (walnuts), and the wood of *Acacia catechu*, which has a high content of condensed tannins [17,18].

Tannin dyes usually lack long-term stability, and their resistance to ageing depends on the combination of originating plant, mordant, and textile, besides environmental parameters [19]. The combination of compositional variability and low fastness of tannin dyes results in complex degradation mechanisms, which are both difficult to study and to reproduce, mainly because they are known to involve the textile matrix and the possible mordant [19]. A complete rationalization of such mechanisms still requires dedicated studies, which are of particular importance in Heritage Science. A detailed knowledge of the composition of tannin dyes in historical textiles can provide insights on the source of the dyestuff, the technique used for its application and the conservation state of the object [20]. These results can also be used to deduce information on the socio-economical context in which the artefacts were produced, as well as the technological level required to produce them.

As natural dyes are usually complex mixtures, in-depth characterization requires the use of separation techniques. Extraction of the dye from the matrix followed by liquid chromatography (LC) with spectroscopic and mass spectrometric detectors is widely used, as it can provide details on the single components of the dyestuff [12,21,22,23]. The main issue in LC analysis of tannins is that polar compounds such as polyphenols are poorly retained by common hydrophobic stationary phases. Nevertheless, embedded-polar group (EPG) stationary phases have proven capable of reliable and efficient separation of the most polar polyphenols, thanks to their ability to operate both in 100% aqueous and in methanol/acetonitrile mobile phases. The efficiency of EPG columns in the analysis of phenolic compounds has already been assessed in the literature [24,25]. Despite these results, scarce information is reported on the use of such stationary phase to analyze tannin dyes.

In the present work, high-performance liquid chromatography (HPLC) with a reversed-phase-amide (RP-amide) EPG stationary phase coupled with high-resolution tandem mass spectrometry was used to characterize the composition of the extracts of wool yarns dyed with natural tannins. This approach allowed us to achieve a suitable retention of the most polar compounds, and an excellent efficiency leading to a thorough characterization of the single components of the extracts.

A set of mock-ups of woolen yarns dyed with different kinds of tannin vegetal sources (gallnut, catechu, and walnut) was prepared. To evaluate the effect of the mordant, samples with aluminum or iron mordants were compared to those without any mordant. Changes in the relative content of the various tannins were evaluated by performing semiquantitative calculations based on integrated peak areas as a function of ageing time. The stability of the dyes was also evaluated by artificial ageing of the samples and by repeating the analyses at different time intervals.

In the end, several historical tapestries were analyzed to assess the nature and state of conservation of their tannin fraction. To the best of our knowledge, this is the first time that RP-amide was used for the analysis of natural tannins dyes in reference textiles and historical tapestries.

## 2. Results

**Figure 1:**
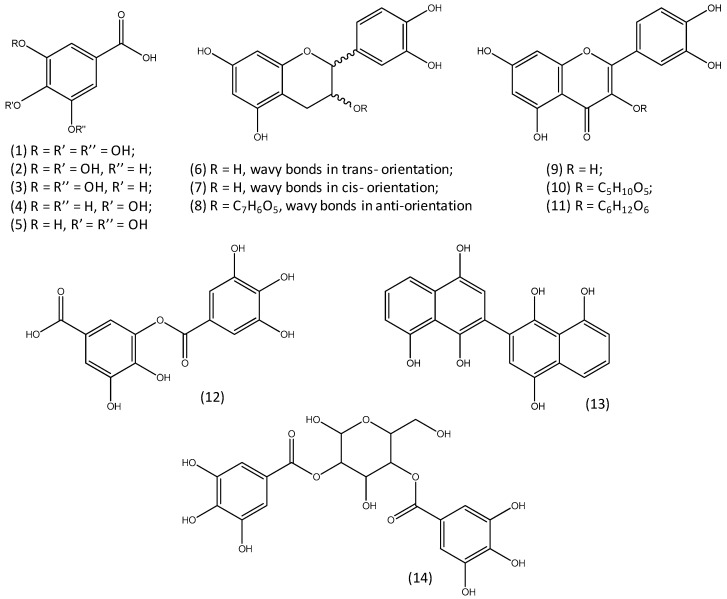
Structures of some representative tannins identified in the reference samples: (1) gallic acid; (2) 3,4-dihydroxybenzoic acid; (3) 3,5-dihydroxybenzoic acid; (4) 4-hydroxybenzoic acid; (5) 3-hydroxybenzoic acid; (6) catechin; (7) epicatechin; (8) gallocatechin; (9) quercetin; (10) quercetin-*O*-pentoside; (11) quercetin-*O*-hexoside; (12) *m*-galloyl-gallate; (13) (2,2′-binaphthalene)-1,1′,4,4′,8,8′-hexaol (juglone dimer); (14) digalloyl-glucose.

Table 1 presents a list of all the compounds that were identified in the reference and historical samples. As will be discussed in the following sections, no qualitative difference in the composition of the extracts was found between the reference samples with or without mordant. Following this observation, reference samples were grouped in Table 1 only according to the dye. For each compound, the chemical formula, molecular weight, and *m/z* value for the molecular ion ([M-H]^-^) are shown. Figure 1 reports the structures of some of the most representative tannins identified in the samples. 

### 2.1. Oak Gallnuts

**Figure 2:**
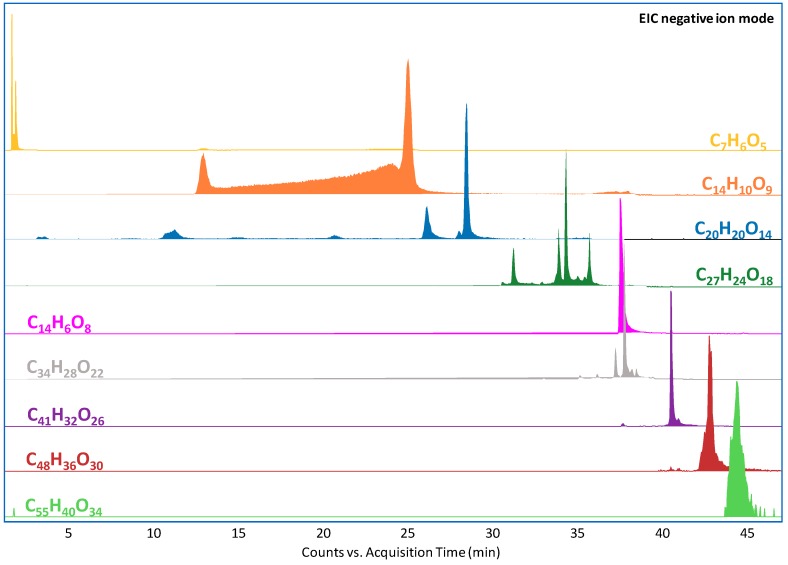
Extracted ion chromatograms from the extract of the nonaged yarn dyed with oak gallnuts without mordant. The molecular formulas correspond to gallic acid (C_7_H_6_O_5_, *m/z* 169.0142), galloyl-gallate (C_14_H_10_O_9_, *m/z* 321.0252), digalloyl-glucose (C_20_H_20_O_14_, *m/z* 483.0780), trigalloyl-glucose (C_27_H_24_O_18_, *m/z* 635.0890), ellagic acid (C_14_H_6_O_8_, *m/z* 300.9990), tetragalloyl-glucose (C_34_H_28_O_22_, *m/z* 787.0999), pentagalloyl-glucose (C_41_H_32_O_26_, *m/z* 939.1109), hexagalloyl-glucose (C_48_H_36_O_30_, *m/z* 1091.1219), and heptagalloyl-glucose (C_55_H_40_O_34_, *m/z* 1243.1328).

The main peaks in all the collected chromatograms for the yarns dyed with gallnuts were attributed to gallic acid and heavier gallotannins. *Meta*- and *para*-galloyl-gallate and a series of galloyl-glycosides were found in all the samples, regardless of the use of mordant. Galloyl-glycosides are the main components of gallnut tannins, where the number of gallic acid units per glucose molecule usually ranges from five to twelve [26]. In the extracts from the dyed yarns, the number of gallic acid molecules per monosaccharide unit generally ranged from two to seven, implying that a slight modification of the tannin profile already took place during the dyeing process. Monogalloyl-glucose was not found in any chromatogram, possibly due to its high polarity. Ellagic acid was also detected in all chromatograms. Figure 2 presents the extracted ion chromatograms (EICs) obtained for the unaged wool yarns without mordant using the *find by formula* algorithm in the data processing software. The peaks of gallic acid, galloyl-gallate, ellagic acid, and all gallotannins are highlighted. Two peaks labeled as galloyl-gallate can be identified, corresponding to the *meta*- and *para*-isomers. Multiple peaks can also be observed for di-, tri-, and tetragalloyl-glucose, corresponding to isomers with different combinations of ester bonds. After 500 h of artificial ageing, few differences were found in the chromatographic profiles in terms of identified compounds. Nevertheless, as will be discussed in the following paragraphs, some significant differences can be observed from a closer look at the relative peak areas of gallotannins.

The wool yarns dyed with gallnuts without any mordant or with an iron-based mordant were also analyzed at four intermediate ageing times, to obtain additional insights into the degradation processes. Integrated areas for each species were normalized by the sample amount and extract volume, to estimate the content of the various gallotannins in each sample. The results are presented in Table 2. The absolute areas of each gallotannin decreased in all cases after 500 h of accelerated ageing. However, the values at intermediate times did not show a monotonic trend, except for a few cases. This result reflects the complex degradation mechanism of these compounds and suggests that the most likely degradation process could be the cleavage of the ester bonds of gallotannins. In fact, such mechanism would cause the content of each gallotannin species to be determined by a sum of a negative contribution (cleavage of the ester bonds to form lighter gallotannins) and a positive contribution (cleavage of the ester bonds from heavier gallotannins), resulting in the observed complex trends.

Table 2 also presents the percentage of gallic acid, calculated by dividing the normalized area of free gallic acid by the total normalized area of all identified compounds. This percentage did not show a monotonic trend in any of the samples. This result shows that, although the hydrolysis of the ester bonds in gallotannins is the most likely degradation mechanism, further degradation processes also take place, leading to the depletion of gallic acid and preventing its accumulation. Literature references show that gallic acid degradation can involve the loss of the hydroxy and/or the carboxy groups [27]. This means that the presence of 3,4-dihydroxy, 4-hydroxy, and 3-hydroxybenzoic acid in the chromatograms of these samples could be due not only to the degradation of wool, but also to the depletion of gallotannins. Degradation products obtained by decarboxylation of gallic acid could not be found in any chromatogram, possibly due to the low molecular weight of such species. 

**Figure 3:**
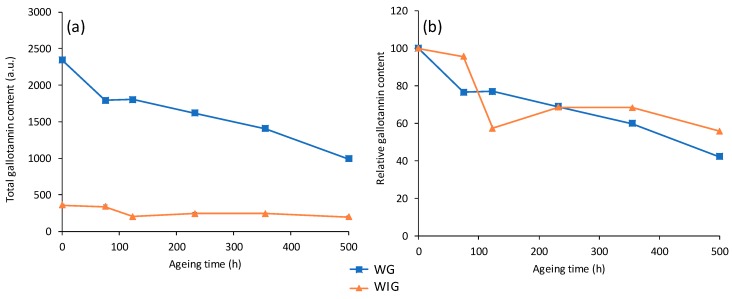
(**a**) Total gallotannins content of samples dyed with oak gallnuts using no mordant (WG) and iron mordant (WIG) as a function of ageing time; (**b**) gallotannins content relative to the nonaged samples as a function of ageing time.

Further information can be obtained by summing the normalized areas of all gallotannins and observing the trend of the total tannin content in the samples as a function of ageing time, as shown in Figure 3a. The total content for the yarns without any mordant is higher than that of the yarns mordanted with iron, at all ageing times. The presence of iron usually has a significant effect on the degradation of the textile matrix, as observed by a morphological point of view in our paper on the field-emission scanning electron microscopy (FESEM) study of the same set of reference yarns [28], as well as by the extent of photo-oxidation of the lipid fraction of wool in our paper on the gas chromatography/mass spectrometry (GC/MS) analysis of the reference set [29]. However, the results obtained in this work suggest that the presence of iron also affects the uptake of gallotannins during the dyeing process. This result should be considered carefully, as the difference in gallotannins content of the two samples could be due to a different extraction efficiency of the Na_2_EDTA/DMF (ethylenediaminetetraacetic acid/dimethylformamide) solution towards the textiles with or without mordant. To rule out the contribution of the extraction strategy and confirm the hypothesis of a different content of gallotannins of the samples, a strong sample treatment entailing acidic hydrolysis with an aqueous HCl/methanol mixture was performed on the nonaged reference yarns with and without iron mordant. The integrated and normalized areas for all gallotannins in the hydrolyzed extracts are presented in Table 2. To compare the efficiencies of the two extraction methods, the ratio between total gallotannins in the samples with and without mordant was calculated in both cases. The value for this ratio in the case of Na_2_EDTA/DMF extraction (6.5) was very close to the one obtained with acidic hydrolysis (6.4), indicating that the extraction efficiency is not altered by the presence of iron.

Figure 3b presents the dependence on the ageing time of the total tannin content for each sample, normalized by the content in the corresponding nonaged sample. The plots in this graph show that artificial ageing had very similar effects on both sets of samples. The tannin content after 500 h of ageing was approximately 45% of the initial one for the wool without any mordant, and approximately 55% for the wool with iron mordant. It appears that in our experimental conditions, the presence of the iron-based mordant only reduces the total gallotannins uptake by the fiber during the dyeing process, but has little influence during artificial ageing.

### 2.2. Acacia catechu 

The chromatographic profiles of the yarns dyed with *Acacia catechu* highlighted the presence of several compounds mostly belonging to the flavonoid family (Table 1). In particular, the molecules identified belong to the following chemical classes: flavan-3-ols (catechin, epicatechin, gallocatechin, profisetidin, and 3,5,7,4′-trihydroxyflavan), flavonols (quercetin and rhamnetin), *O*-methylated flavonols (kaempferide), flavanones (eriodictyol), flavanonols (taxifolin), cinnamic acids (caffeic and 3,4-dimethoxycinnamic acids), and benzoic acid (gallic, 3,4-dihydroxybenzoic, 4-hydroxybenzoic, and 3-hydroxybenzoic acids). The presence of these compounds in catechu extracts has been documented in the literature [30]. No qualitative differences were disclosed by comparing samples with different mordants or after artificial ageing.

**Figure 4:**
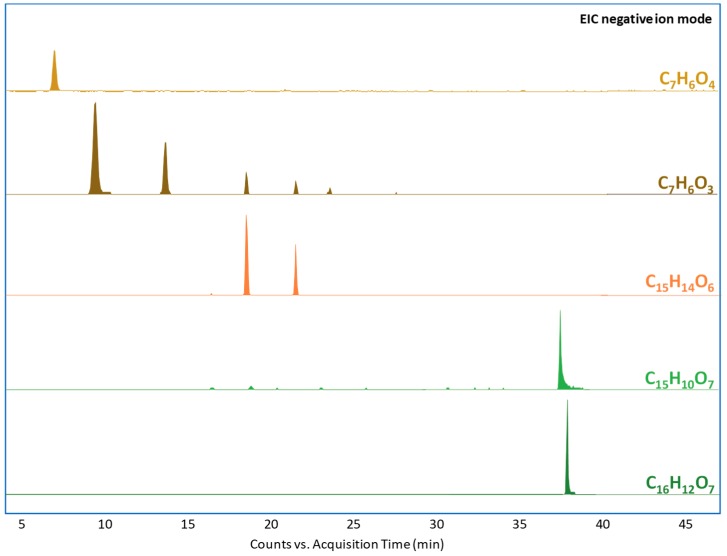
Extracted ion chromatograms from the extract of the nonaged yarn dyed with *Acacia catechu* and without mordant. The molecular formulas correspond to 3,4-dihydroxybenzoic acid (C_7_H_6_O_4_, *m/z* 153.0193), 4-hydroxybenzoic acid and 3-hydroxybenzoic acid (C_7_H_6_O_3_, *m/z* 137.0244), catechin and epicatechin (C_15_H_14_O_6_, *m/z* 289.0718), quercetin (C_15_H_10_O_7_, *m/z* 301.0354), and rhamnetin (C_16_H_12_O_7_, *m/z* 315.0510).

The EICs of the main compounds identified in the nonaged wool yarns without any mordant are reported in Figure 4. Catechin and epicatechin are the main species present in *Acacia catechu* [31]. Since these two species are epimers (like profisetidin and 3,5,7,4′-trihydroxyflavan), their distinction was based on the elution order rather than their tandem mass spectra [32].

**Figure 5:**
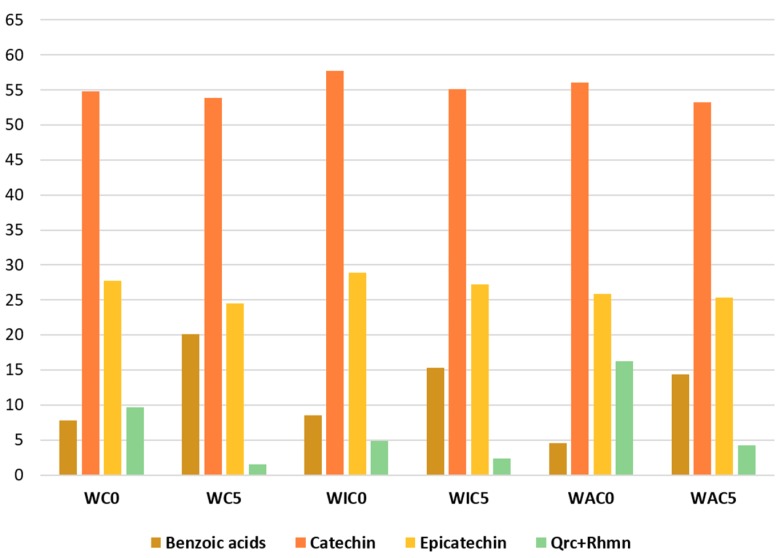
Histograms of percentage peak areas of benzoic acids (3,4-dihydroxybenzoic acid + 4-hydroxybenzoic acid + 3-hydroxybenzoic acid), catechin, epicatechin, quercetin + rhamnetin in yarns dyed with *Acacia catechu*; samples are labelled according to the following code: W = wool, C = dyed with *Acacia catechu*, I = iron-mordant, A = aluminum mordant, 0 = unaged, 5 = aged for 500 h. A full list of the samples labels is provided in Table 3.

Chromatographic areas integrated for each species were converted into percentages, and the results are presented in Figure 5. The areas of hydroxy- and dihydroxybenzoic acids (4-hydroxybenzoic acid, 3-hydroxybenzoic acid, and 3,4-dihydroxybenzoic acid), and those of the flavonoids (quercetin and rhamnetin) were summed to aid visual interpretation.

As expected, ageing results in the decrease of catechin, epicatechin, quercetin, and rhamnetin and the increase of benzoic acids [27]. After 500 h of artificial ageing, flavan-3-ols slightly decreased, while flavonols showed a more enhanced depletion, evidencing a different resistance to degradation. Artificial ageing caused an increase in the extracts content of benzoic acids, and the changes were more significant than those observed for the yarns dyed with gallnuts. Similar variations were observed both in presence and in absence of mordants, revealing that the mordant does not play a fundamental role in the degradation of flavan-3-ols and flavonols within catechu extracts in the adopted conditions. In this case, the presence of the mordant does not seem to affect the total dyeing molecules uptake by the fiber, different from what was observed for oak gallnut dye bath.

### 2.3. Juglans regia

The extracts of yarns dyed with *Juglans regia* contain several classes of compounds (Table 1) in accordance with the composition reported in the literature: flavonols (quercetin), flavonol *O*-glycosides (quercetin 3-galactoside, quercetin 3-arabinoside, quercetin 3-xyloside and quercetin 3-rhamnoside), cinnamic acids (caffeic acid and 3,4-dimethoxycinnamic acid), hydroxycinnamic acids and esters (*p*-coumaric acid, *p*-coumaroylquinic acid, 3-*O*-caffeoylquinic acids and 5-*O*-caffeoylquinic acids), benzoic acids (gallic acid, 3,4-dihydroxybenzoic acid, 4-hydroxybenzoic acid, 3-hydroxybenzoic acid, and vanillic acid), benzoic esters (ethyl gallate), as well as ellagic acid [33,34,35]. Juglone (5-hydroxy-1,4-naphthoquinone) is known as the marker compound of *Juglans* species [36], but it is absent in all the reference yarns analyzed. This is not surprising, considering that juglone is not generally found in walnut extracts due to its high tendency to undergo reversible redox reactions with the simultaneous formation of free radicals [37]. Moreover, its solubility in several solvents is limited [33]. Interestingly, one peak whose molecular ion corresponds to that of a dimeric form of juglone, namely (2,2′-binaphthalene)-1,1′,4,4′,8,8′-hexaol, was found in all the samples except for the aged wool yarn that was dyed without mordant. The formation of this compound has already been observed from juglone after chemical treatment [38]. Even if our experimental conditions differ from those reported in the literature, the initial content of juglone present in the extract might have been converted in this dimeric form.

**Figure 6:**
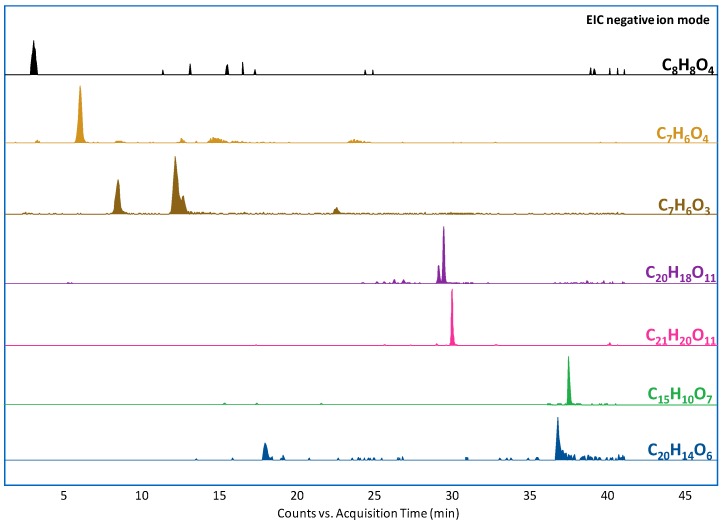
Extracted ion chromatograms from the extract of the nonaged yarn dyed with walnut and without mordant. The molecular formulas correspond to vanillic acid (C_8_H_8_O_4_, *m/z* 167.0350), 3,4-dihydroxybenzoic acid (C_7_H_6_O_4_, *m/z* 153.0193), 4-hydroxybenzoic acid and 3-hydroxybenzoic acid (C_7_H_6_O_3_, *m/z* 137.0244), quercetin 3-galactoside and quercetin 3-rhamnoside (C_20_H_18_O_11_, *m/z* 433.0776), quercetin 3-arabinoside and quercetin 3-xyloside (C_21_H_20_O_11_, *m/z* 447.0933), and juglone dimer (C_20_H_14_O_6_, *m/z* 349.0718, tentative attribution).

**Figure 7:**
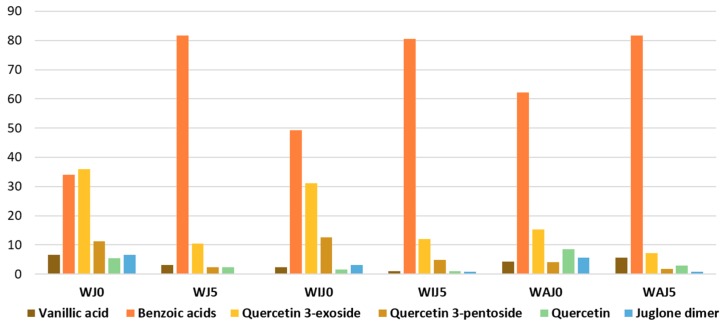
Histogram of percentage MS areas of vanillic acid, benzoic acids (3,4-dihydroxybenzoic acid + 4-hydroxybenzoic acid + 3-hydroxybenzoic acid), quercetin 3-hexoside (quercetin 3-galactoside + quercetin 3-rhamnoside) and quercetin 3-pentoside (quercetin 3-arabinoside + quercetin 3- xyloside), quercetin and juglone dimer (tentative attribution) in yarns died with *Juglans regia* nuts; samples are labelled according to the following code: W = wool, J = dyed with *Juglans regia*, I = iron-mordant, A = aluminum mordant, 0 = nonaged, 5 = aged for 500 h. A full list of the samples labels is provided in Table 3.

The extract ion chromatograms of the most abundant compounds identified in the samples and the histograms reporting the composition as percentage areas are provided in Figure 6 and Figure 7, respectively. The area of hydroxy- and dihydroxybenzoic acids (4-hydroxybenzoic, 3-hydroxybenzoic, and 3,4-dihydroxybenzoic acids), quercetin 3-hexosides (quercetin 3-galactoside and quercetin 3-rhamnoside), and quercetin 3-pentosides (quercetin 3-arabinoside and quercetin 3-xyloside) were summed to aid visual interpretation. 

In all the samples, the amount of quercetin 3-pentosides, quercetin 3-hexosides, quercetin, juglone dimer, and vanillic acid decrease after ageing, while that of benzoic acids increased drastically. Flavonols in *Juglans* are more sensitive to photo-oxidation with respect to other flavonoids, as already observed for *Acacia catechu*. Considering that quercetin 3-hexosides, quercetin 3-pentosides, and quercetin are the main source of tannins in walnut extracts, a strong increase of benzoic acid concentration with ageing is expected. The increase is particularly intense for the sample without any mordant, possibly due to the lack of formation of flavonoid–metal complexes that may stabilize them. Contrarily to what was observed for the yarns dyed with oak gallnuts and catechu, benzoic acids were the main components even before artificial ageing, also being metabolites present in the plant species [39]. Thus, a straightforward interpretation on the variation of concentration of benzoic acids in relation to ageing was not possible.

### 2.4. Historical Samples

The procedure adopted for the analysis of reference materials succeeded in characterizing the composition of all the 14 historical samples. In almost all of them the main compounds were gallic acid, hydroxybenzoic acids (3,4-dihydroxybenzoic, 3,5-dihydroxybenzoic, 4-hydroxybenzoic, and 3-hydroxybenzoic acids), and ellagic acid. Gallotannins (digalloyl-glucose, *p*-galloyl-gallate and *m*-galloyl-gallate) were detected only in *Abbondanza 2*, suggesting that oak gallnuts with an iron mordant are the most likely candidates as dyeing materials. The high content of benzoic acids is indicative that tannin dyes were responsible for the yarn coloration, but the lack of surviving flavonoids does not allow us determining which specific source of tannins was used.

## 3. Discussion

The different behavior of natural tannin dyes after artificial ageing were outlined from the analysis of a wide set of reference samples. Gallotannins and ellagitannins were the main components of oak gallnuts, and their content was drastically reduced using iron-based mordant in the dyeing process, even before artificial ageing. The total content of gallotannins decreased significantly after ageing, while there was a slight increase in the content of hydroxybenzoic acids. The main components of catechu were catechin and epicatechin, together with other flavonoids such as quercetin and rhamnetin. Ageing of these tannins led to a drastic increase in the content of hydroxybenzoic acids and to a significant decrease in the flavonoids content, although catechin and epicatechin were less affected by photo-oxidation than other species. Finally, the main components of walnut tannins were quercetin and its glycosides, as well as phenolic acids such as vanillic acid. Walnut samples also showed a high content of hydroxybenzoic acids both before and after ageing.

In most cases, the use of alum or iron mordant did not appear to influence the resistance of the dyes to ageing. Two exceptions were found to this. First, the use of iron mordant with gallotannins reduced the total tannin uptake by the wool yarns already during the dyeing process. Second, the presence of alum or iron mordants in the yarns dyed with walnut was associated with less extensive degradation of the tannins, suggesting that the formation of metal–flavonoid complexes increase the stability of the dye. These results require a more detailed investigation, as the data obtained in the present work are not sufficiently supported to outline a consistent trend in the effects of the mordant on the degradation of tannin dyes.

All historical samples showed extensive degradation, as gallic acid, ellagic acid, and hydroxybenzoic acids were the only compounds that could be detected. Only one sample from the tapestry *L’abbondanza* showed peaks that were attributed to gallotannins, suggesting that this sample was dyed using oak gallnuts. Given that hydroxybenzoic acids can derive from the degradation of all considered tannin dyes, it was not possible to simply establish the plant source for the dyes in any of the other samples.

The present work highlights the efficacy of HPLC coupled with high-resolution mass spectrometry detection for the thorough characterization of tannin dyes in textiles. Thanks to the use of an embedded polar group stationary phase, we achieved good retention of very polar compounds, such as hydroxybenzoic acids, whose presence in both the reference and historical samples allowed us to discuss the effects of ageing on natural tannin dyes of different origins. Further studies are required to improve our knowledge on the degradation mechanisms of tannin dyes, especially regarding the effect of the presence of mordants during the dyeing process, and the influence of environmental parameters such as temperature and relative humidity.

## 4. Materials and Methods 

### 4.1. Chemicals

The solvents used for the HPLC analysis and sample pretreatment were: water (LC-MS grade, Sigma Aldrich, St. Louis, MO, USA), acetonitrile (ACN; LC-MS grade, Sigma Aldrich), formic acid (FA, 98%; J.T. Baker, Waltham, MA, USA), dimethylformamide (DMF; 99.8% purity, J.T. Baker), ethylenediaminetetraacetic acid disodium salt (Na_2_EDTA·H_2_O; Fluka, Charlotte, NC, USA), methanol (MeOH, HPLC grade; Sigma-Aldrich), and hydrochloric acid (HCl 37%; Merck, Darmstadt, Germany). PTFE filters (4 mm thickness and 0.45 μm pore diameter) were used for the purification prior to HPLC injection.

### 4.2. Reference Samples

Thirty-two reference wool samples were analyzed. The sample sets were prepared during the *Short Life of Tannins* project (http://www.scich.it/vat), and consist of wool yarns dyed with walnut (*Juglans regia*), catechu (*Acacia catechu*) and oak gallnuts, using aluminum and iron (II) salts as mordants. Samples without any dye or mordant were also prepared to compare the results. A list of all the samples is presented in Table 3, where a label has also been assigned to each wool yarn. Raw gall, walnut, and catechu were from Kremer Pigmente (Aichstetten, Germany). Cream of tartar (potassium hydrogen tartrate) and iron (II) sulfate were from Carlo Erba (Milan, Italy), while alum (aluminium potassium sulfate) was purchased from Zecchi Colori belle arti restauro (Florence, Italy). Wool was purchased from Campolmi Filati (Florence, Italy). The ageing of the samples was performed using a SolarBox 1500e RH weathering chamber (CO.FO.ME.GRA., Italy), equipped with a xenon lamp and a borosilicate glass outdoor filter ultraviolet (UV) S201 (280 nm). Ageing was performed with an irradiance of 500 W/m^2^ at 35 °C and 50% relative humidity. As reported in Table 3, most of the samples were analyzed after 500 h of ageing. Wool yarns dyed with gallnuts, using no mordant and iron-based mordant, were also analyzed at four intermediate times.

### 4.3. Historical Samples

Fourteen historical samples collected from four Florentine manufactory tapestries dated to 15^th^, 16^th^ century were analyzed. The samples were kindly provided by OPD (*Opificio delle Petre Dure*, Florence, Italy) during restoration campaigns. Eight samples were taken from “*L’assalto finale a Gerusalemme*” (*The final attack to Jerusalem*, Ger 1-8), three from “*Giuseppe fugge dalla moglie di Putifarre*” (*Joseph flees from Potiphar’s wife*, Giu 1-3), two from “*L’abbondanza*” (*The plenitude*, Abb 1 and 2), and one from “*Giacobbe benedice i figli di Giuseppe*” (*Jacob blesses Joseph’s sons*, Gia).

### 4.4. Sample Pretreatment

All the reference materials and historical samples were subjected to a mild sample pretreatment [40]: addition of 500 μL of extracting solution (1:1 *v/v* mixture of 0.1% Na_2_EDTA in H_2_O and DMF) to c.a. 5 mg of sample, sonication for 1 h at 60 °C, filtration of the supernatant with PTFE syringe filters. 

In order to determine the exact content of gallotannins, a stronger extraction method allowing the hydrolysis of poly-gallotannins to gallic acid was employed. The method is based on an acidic hydrolysis [41]: addition of 500 μL of HCl/MeOH solution (1:30) to c.a. 5 mg of sample, sonication for 1 h at 60 °C, drying under nitrogen flow, re-dissolution in 500 μL of extracting solution, filtration of the supernatant with PTFE syringe filters.

### 4.5. High-Performance Liquid Chromatography-Tandem Mass Spectrometry (HPLC-MS^2^)

All experiments were performed with an HPLC 1200 Infinity chromatographic system, coupled with a 6530 Infinity Q-ToF tandem mass spectrometer by a Jet Stream ESI interface (Agilent Technologies, Santa Clara, California, USA). Separation was achieved using an Ascentis Express RP-amide column (10 cm x 2.1 mm, particle diameter 2.7 µm, Sigma-Aldrich) with an Ascentis Express RP-amide guard column (5 mm x 2.1 mm, particle diameter 2.7 µm, Sigma-Aldrich). Eluents were water (A) and acetonitrile (B), both with 0.3% (*v/v*) formic acid. Mobile phase flow rate was 0.4 mL/min. Elution was performed with the following composition gradient: 0–3.75 min at 100% (A); 3.75–19.50 min from 100% to 89% (A); 19.50–27.75 min from 89% to 79% (A); 27.75–44.25 min from 79% to 60% (A); 44.25–50.25 min from 60% to 37% (A); 50.25–51.00 min from 37% to 0% (A); 51.00–52.00 min at 0% (A); 52.00–54.00 return to 100% (A). Re-equilibration time was 10 min.

N_2_ (purity > 98%) was used as drying and sheath gas for the ESI interface. Ionization was operated in negative ion mode in the following conditions: drying gas temperature 350 °C, flow 10 L/min, capillary voltage 4.5 kV; nebulizer gas pressure 35 psig; sheath gas temperature 375 °C, flow 11 L/min. High resolution MS and MS/MS acquisition range was set from 100 to 1700 *m/z* at a scan rate of 1.04 spectra/s. Collision-induced fragmentation was performed using nitrogen (99.999% purity) as collision gas working at 20 V potential. Calibration of the mass axis was performed daily using the Agilent HP0321 tuning mix (Agilent Technologies) prepared in acetonitrile.

### 4.6. Data Processing

Chromatograms obtained from HPLC-MS^2^ analyses were processed using MassHunter Workstation (v. B.04.00, Agilent Technologies). Extracted ion chromatograms (EIC) were obtained using the *find by formula* algorithm implemented in the software; the identification threshold was set at 2 ppm tolerance. Identification of the compounds was based on their MS and MS/MS spectra, and by comparison with literature references [11]. Reproducibility of the procedure was determined by extracting and analyzing three aliquots of the same yarn and integrating the peak of gallic acid for each replicate. A relative standard deviation of 10% was obtained.

## Figures and Tables

**Table 1:** List of identified compounds in reference and historical samples. For each compound, the chemical formula, molecular weight, molecular ions, and retention times are presented. In the case of undistinguished isomers, the retention time reported is relative to the first one eluted. The labels G, J, and C indicate reference samples dyed with gallnuts, walnut, and catechu, respectively.

Compound	Formula	MW (a.m.u.)	*m/z*	G	J	C	Ger1	Ger2	Ger3	Ger4	Ger5	Ger6	Ger7	Ger8	Abb1	Abb2	Gia	Giu1	Giu2	Giu3
Gallic acid	C_7_H_6_O_5_	170.12	169.014	x	x	x	x	x	x	x	x	x	x			x		x	x	x
Vanillic acid	C_8_H_8_O_4_	168.14	167.023							x				x			x		x	
3,4-dihydroxybenzoic acid	C_7_H_6_O_4_	154.12	153.019	x	x	x	x		x		x	x	x				x			
3,5-dihydroxybenzoic acid	C_7_H_6_O_4_	154.12	153.019	x			x		x	x	x	x	x			x	x	x	x	x
4-hydroxybenzoic acid	C_7_H_6_O_3_	138.12	137.017		x	x	x	x	x	x	x	x	x	x	x	x	x	x	x	x
3-hydroxybenzoic acid	C_7_H_6_O_3_	138.12	137.017		x	x	x	x	x	x	x	x	x	x	x	x	x	x	x	x
3,4-dimethoxycinnamic acid	C_11_H_12_O_4_	208.07	207.055			x														
Caffeic acid	C_9_H_8_O_4_	180.16	179.081			x														
p-galloylgallate	C_14_H_10_O_9_	322.03	321.016	x												x				
Ethyl gallate	C_9_H_10_O_5_	198.17	197.045		x															
Catechin	C_15_H_14_O_6_	290.26	289.06			x														
Digalloyl-glucose	C_20_H_20_O_14_	484.18	483.065	x												x				
Gallocatechin	C_15_H_14_O_7_	306.07	305.053			x														
Profisetidin	C_15_H_14_O_5_	274.08	273.064			x														
p-coumaroylquinic acid	C_16_H_18_O_8_	338.31	337.078		x															
m-galloylgallate	C_14_H_10_O_9_	322.03	321.01	x												x				
Epicatechin	C_15_H_14_O_6_	290.26	289.06			x														
p-coumaric acid	C_9_H_8_O_3_	164.04	163.029		x															
3,5,7,4′-Trihydroxyflavan	C_15_H_14_O_5_	274.08	273.064			x														
Trigalloyl-glucose	C_27_H_24_O_18_	636.28	635.076	x																
Catechin-3-O-gallate	C_22_H_18_O_10_	442. 37	441.07			x														
Taxifolin	C_12_H_12_O_7_	304.25	303.039			x														
Tetragalloyl-glucose	C_34_H_28_O_22_	788.39	787.080	x																
Quercetin 3-galactoside	C_21_H_20_O_12_	464.38	463.072		x															
Ellagic acid	C_14_H_6_O_8_	302.2	300.987	x	x			x	x	x	x	x		x	x	x			x	x
Eriodictyol	C_15_H_12_O_6_	288.25	287.043			x														
Quercitin 3-pentoside	C_20_H_18_O_11_	434.35	433.061		x															
Kaempferide	C_16_H_12_O_6_	300.26	299.042			x														
Quercetin 3-hexoside	C_21_H_20_O_11_	448.38	447.077		x															
Pentagalloyl-glucose	C_41_H_32_O_26_	940.49	939.091	x																
3-O-caffeoylquinic acids	C_16_H_18_O_9_	354.31	353.089		x															
Hexagalloyl-glucose	C_48_H_36_O_30_	1092.58	1091.096	x																
Eptagalloyl-glucose	C_55_H_40_O_34_	1244.7	1243.102	x																
5-O-caffeoylquinic acids	C_16_H_18_O_10_	354.31	353.089		x															
Quercetin	C_15_H_10_O_7_	302.24	301.023		x	x														
Rhamnetin	C_16_H_12_O_7_	316.26	315.039			x														
Juglone dimer (tentative attribution)	C_20_H_14_O_6_	350.07	349.056		x															

**Table 2:** Normalized peak areas of all identified compounds in the extracts of yarns dyed with oak gallnuts using either no mordant or iron mordant, as a function of ageing time. The total gallotannins content and the percentage of free gallic acid with respect to total identified compounds are also presented for each sample.

		Integrated and Normalized Areas (10^6^)						
	Ageing Time (h)	0	0 (HCl)	75	123	232	355	500
**No mordant**	hydroxybenzoic acids	2	0	23	10	12	16	19
	gallic acid	586	308	311	265	468	379	295
	galloyl-gallate	322	62	303	237	226	201	161
	digalloyl-glucose	174	273	173	175	150	112	87
	trigalloyl-glucose	305	274	295	319	163	212	148
	tetragalloyl-glucose	374	395	324	355	246	243	138
	pentagalloyl-glucose	466	469	315	374	289	209	129
	hexagalloyl-glucose	102	149	74	70	68	46	33
	heptagalloyl-glucose	15	27	0	11	9	6	4
	Total gallotannins	2344	1957	1795	1806	1619	1408	995
	Free gallic acid (% of TOT)	25.0	15.7	17.3	14.7	28.9	26.9	29.6
**Iron mordant**	hydroxybenzoic acids	0	0	0	0	2	2	2
	gallic acid	153	108	114	72	86	84	72
	galloyl-gallate	58	2	49	44	42	50	45
	digalloyl-glucose	12	92	14	7	12	10	11
	trigalloyl-glucose	24	21	30	16	24	20	17
	tetragalloyl-glucose	36	31	51	20	31	26	21
	pentagalloyl-glucose	54	52	59	31	35	34	23
	hexagalloyl-glucose	21	0	25	15	16	21	10
	eptagalloyl-glucose	0	0	0	0	0	0	0
	Total gallotannins	358	305	342	206	246	245	200
	Free gallic acid (% of TOT)	42.6	35.3	33.3	35.1	35.1	34.3	36.1

**Table 3:** List of all the analyzed samples. Labels for each sample are presented in the rightmost column.

**No dye**		
No mordant	Unaged	W0
	Aged 500 h	W5
Aluminium	Unaged	WA0
	Aged 500 h	WA5
Iron	Unaged	WI0
	Aged 500 h	WI5
**Oak gallnuts**		
No mordant	Unaged	WG0
	Aged 75 h	WG1
	Aged 123 h	WG2
	Aged 232 h	WG3
	Aged 355 h	WG4
	Aged 500 h	WG5
Aluminium	Unaged	WAG0
	Aged 500 h	WAG5
Iron	Unaged	WIG0
	Aged 75 h	WIG1
	Aged 123 h	WIG2
	Aged 232 h	WIG3
	Aged 355 h	WIG4
	Aged 500 h	WIG5
**Catechu (*Acacia catechu*)**		
No mordant	Unaged	WC0
	Aged 500 h	WC5
Aluminium	Unaged	WAC0
	Aged 500 h	WAC5
Iron	Unaged	WIC0
	Aged 500 h	WIC5
**Walnut (Juglans regia)**		
No mordant	Unaged	WJ0
	Aged 500 h	WJ5
Aluminium	Unaged	WAJ0
	Aged 500 h	WAJ5
Iron	Unaged	WIJ0
	Aged 500 h	WIJ5
